# Adipose Tissue Steroid Receptor RNA Activator 1 (SRA1) Expression Is Associated with Obesity, Insulin Resistance, and Inflammation

**DOI:** 10.3390/cells10102602

**Published:** 2021-09-30

**Authors:** Shihab Kochumon, Hossein Arefanian, Sardar Sindhu, Steve Shenouda, Reeby Thomas, Fahd Al-Mulla, Jaakko Tuomilehto, Rasheed Ahmad

**Affiliations:** 1 Department of Immunology & Microbiology, Dasman Diabetes Institute, Dasman 15462, Kuwait; shihab.kochumon@dasmaninstitute.org (S.K.); hossein.arefanian@dasmaninstitute.org (H.A.); steve.shenouda@dasmaninstitute.org (S.S.); reeby.thomas@dasmaninstitute.org (R.T.); 2 Animal and Imaging Core Facilities, Dasman Diabetes Institute, Dasman 15462, Kuwait; sardar.sindhu@dasmaninstitute.org; 3 Department of Genetics and Bioinformatics, Dasman Diabetes Institute, Dasman 15462, Kuwait; fahd.almulla@dasmaninstitute.org; 4 Department of Public Health, University of Helsinki, 00100 Helsinki, Finland; jaakko.tuomilehto@thl.fi; 5 Public Health Promotion Unit, Diabetes Research Group, Finnish Institute for Health and Welfare, King Abdulaziz University, Jeddah 21589, Saudi Arabia

**Keywords:** steroid receptor RNA activator 1/*SRA1*, adipose tissue, obesity, type 2 diabetes, insulin resistance, inflammation

## Abstract

Steroid receptor RNA activator 1 (*SRA1*) is involved in pathophysiological responses of adipose tissue (AT) in obesity. In vitro and animal studies have elucidated its role in meta-inflammation. Since *SRA1* AT expression in obesity/type 2 diabetes (T2D) and the relationship with immune-metabolic signatures remains unclear, we assessed AT *SRA1* expression and its association with immune–metabolic markers in individuals with obesity/T2D. For this, 55 non-diabetic and 53 T2D individuals classified as normal weight (NW; lean), overweight, and obese were recruited and fasting blood and subcutaneous fat biopsy samples were collected. Plasma metabolic markers were assessed using commercial kits and AT expression of *SRA1* and selected immune markers using RT-qPCR. *SRA1* expression was significantly higher in non-diabetic obese compared with NW individuals. *SRA1* expression associated with BMI, PBF, serum insulin, and HOMA-IR in the total study population and people without diabetes. *SRA1* associated with waist circumference in people without diabetes and NW participants, whereas it associated inversely with HbA1c in overweight participants. In most study subgroups AT *SRA1* expression associated directly with *CXCL9, CXCL10, CXCL11, TNF-α, TGF-β, IL2RA*, and *IL18*, but inversely with *CCL19* and *CCR2*. *TGF-β/IL18* independently predicted the *SRA1* expression in people without diabetes and in the total study population, while *TNF-α/IL-2RA* predicted *SRA1* only in people with diabetes. *TNF-α* also predicted *SRA1* in both NW and obese people regardless of the diabetes status. In conclusion, AT *SRA1* expression is elevated in people with obesity which associates with typical immunometabolic markers of obesity/T2D, implying that *SRA1* may have potential as a biomarker of metabolic derangements.

## 1. Introduction

SRA1 is a gene that encodes steroid receptor RNA activator 1 (SRA1) [1]. SRA1 was initially characterized as an intergenic long non-coding RNA (lncRNA)that functions as an RNA coactivator of nuclear receptors to enhance steroid receptor-dependent gene expression [1]. It was reported that SRA1 can modulate gene transcription in the cell- and system-specific manners [1,2,3,4]. SRA1 can be recruited to DNA through interactions with other proteins that bind either directly or indirectly to DNA and serves as a natural organizer, regulating multiple physiological functions that govern the epigenetic modifications, modulation of chromatin modification, and gene expressions [5,6,7].

The role of SRA1 and its coactivators in the regulation of mammary gland development, myocyte, and adipocyte differentiation, steroidogenesis, tumorigenesis, hepatic steatosis, stem cell function, lipid metabolism, glucose homeostasis, and insulin sensitivity in adipocytes has been well documented [1,8,9]. Indeed, aberrant SRA1 expression and recent mutant variants have been identified in several clinical samples [4,5,7,8,9,10,11,12]. Higher expression levels of SRA1 in the human liver, muscle tissues, and in white and brown adipose tissues as key organs in the regulation of metabolic homeostasis compared to other tissues have been reported [1,8,13]. In fact, SRA1 is now known to be associated with several diseases, including obesity, cardiovascular diseases, polycystic ovary syndrome (PCOS), laryngeal squamous cell carcinoma, and breast cancer [3,8,9,10,13,14,15,16,17].

Obesity results from an imbalance between energy intake and expenditure, in which the excess energy is stored as triglyceride in white adipose tissue with both increased fat cell size (hypertrophy) and number (hyperplasia) [18]. Obesity induces a state of chronic, low-grade inflammation in fat that is accompanied by the local secretion of cytokines and chemokines, causing attenuation of insulin action [19,20,21,22,23]. Obesity is closely associated with a number of diseases including type 2 diabetes (T2D), cardiovascular disease, hypertension, dementia, and certain cancers [24,25]. Adipocytes function both as reservoirs of fuel and as endocrine cells, secreting adipokines such as leptin, adiponectin, interleukin-6 (IL-6), and tumor necrosis factor-α (TNF-α) to regulate whole-body energy metabolism and glucose homeostasis [26,27].

Based on our knowledge, the level of SRA1 expression in the AT in obesity and its association with insulin resistance and metabolic inflammation in humans in a clinical setting are poorly understood. Therefore, in this study, we aimed at investigating the levels of expression of SRA1 in the subcutaneous AT and determined its association with the inflammatory and metabolic markers in overweight/obese individuals, with or without T2D.

## 2. Materials and Methods

### 2.1. Study Population and Anthropometric Measurements

The study comprised 108 participants including 55 non-diabetic (8 NW, 19 overweight, and 28 obese), and 53 T2D (4 NW, 13 overweight, and 36 obese) individuals. In accordance with the ethical guidelines of the Declaration of Helsinki which is approved by the ethics committee of Dasman Diabetes Institute, Kuwait, (grant number RA 2010-003), each study participant submitted written informed consent for participation in the study. The exclusion criteria included chronic diseases of the lung, heart, kidney, or liver; hematologic disorders; pregnancy; immune dysfunction; type 1 diabetes, or malignancy, as previously described [28].

### 2.2. Anthropometric Measurements

Height and weight were measured using calibrated, portable electronic weighing scales and portable, inflexible height-measuring bars; waist and hip circumferences were measured using constant-tension tape. Body mass index (BMI, kg/m^2^) was calculated as the weight (kg)/height (m^2^) and participants were allocated in different categories as lean: BMI < 25 kg/m^2^; overweight: 25 ≤ BMI < 30 kg/m^2^, and obese: BMI ≥ 30 kg/m^2^. IOI353 Body Composition Analyzer (Jawon Medical, South Korea) was used to examine the whole-body composition and percentage of body fat (PBF). Fasting plasma glucose (FPG) and serum insulin measurements were used in the updated homeostasis model assessment (HOMA) index to evaluate insulin resistance (HOMA-IR), and insulin sensitivity [29].

### 2.3. Collection of Subcutaneous Adipose Tissue

Human adipose tissue biopsies (about 0.5 g) were collected from the abdominal subcutaneous fat pad just lateral to the umbilicus using standard sterile surgical methods as previously described [30]. Briefly, the periumbilical area was swabbed with ethanol and then locally anesthetized with 2% lidocaine (2 mL, Fresenius Kabi, LLC., Lake Zurich, IL, USA). Through a small superficial skin incision (0.5 cm), fat tissue was collected. After removal, biopsy tissue was further incised into smaller pieces (~50–100 mg) in RNAlater (Sigma-Aldrich Chemie GmbH, Taufkirchen, Germany) for RNA extraction, and then stored at −80 °C until use [31].

### 2.4. Measurement of Metabolic Markers

Peripheral blood was collected from overnight-fasted individuals and analyzed for FPG, fasting insulin, lipid profile, and HbA1c. FPG and lipid profiles including plasma triglycerides (TGL), low-density lipoproteins (LDL), high-density lipoproteins (HDL), and cholesterol levels were measured using Siemens Dimension RXL chemistry analyzer (Diamond Diagnostics, Holliston, MA, USA). HbA1c was measured using a Variant device (BioRad, Hercules, CA, USA).

### 2.5. RNA Extraction, cDNA Synthesis, and Detection of SRA1 and Inflammatory Markers by RT-qPCR

Total RNA was extracted from the adipose tissue samples using an RNeasy kit (Qiagen, Valencia, CA., USA) following the manufacturer’s protocol. The first-strand cDNA was synthesized from 0.5 µg RNA using a High-Capacity cDNA Reverse Transcription kit (Applied Biosystems, CA, USA) as previously described [30]. Real-time qRT-PCR was performed as we described previously [30]; cDNA samples (50 ng) were amplified using TaqMan Gene Expression Master Mix (Applied Biosystems, CA, USA) and gene-specific 20 × TaqMan gene expression assays containing forward and reverse primers (Table 1) and a target-specific TaqMan MGB probe labeled with FAM dye at the 5′-end and NFQ-MGB at the 3′-end of the probe using a 7500 Fast Real-Time PCR System (Applied Biosystems, CA, USA). Each cycle involved denaturation (15 s at 95 °C), annealing/extension (1 min at 60 °C) after UDG (2 min at 50 °C) and AmpliTaq gold enzyme (10 min at 95 °C) activation. Relative gene expression to control, lean AT, was calculated using the comparative Ct method as we previously described [32]. Results were normalized to GAPDH and expressed as mean ± standard error (SD) values relative to controls [33].

### 2.6. Statistical Analysis

Statistical analysis was performed using GraphPad Prism software (GraphPad, La Jolla, CA, USA) and SPSS for Windows version 19.01 (IBM SPSS Inc., Chicago, IL, USA). Unless otherwise indicated, data were shown as mean ± SD values. A non-parametric Mann–Whitney U test was used to compare means between groups. Spearman correlation and stepwise multivariable regression analysis were performed to determine associations between different variables. For all analyses, a *p* value < 0.05 was considered significant. Standard multivariable linear regression by the Enter method was used; variables that significantly correlated with SRA were selected as predictor variables and were entered simultaneously to generate the model. The *F*-test was used to assess whether the set of entered independent variables collectively predicted the dependent variable. *R*-squared was used to determine how much variance in the dependent variable could be accounted for by the set of independent variables. The *t* test, *p* value, and beta coefficients (β-value) were used to determine the significance and the magnitude of prediction for each independent variable, respectively.

## 3. Results

### 3.1. Demographic and Clinical Characteristics of the Study Population

The characteristics of 108 individuals that participated in this study are detailed in Table 2. Significant differences were observed between non-diabetic (*n* = 55) and diabetic (*n* = 53) participants for FPG, fasting insulin, TG, HbA1c, and HOMA-IR. No significant differences were found between non-diabetic vs. diabetic participants, comparing for weight, height, BMI, waist circumference, hip circumference, waist/hip ratio (WHR), and PBF. However, these parameters, except for height, differed significantly between lean, overweight, and obese non-diabetic or diabetic participants, as expected. The study participants were 34–58 years old, with a significant age difference between people with and without diabetes, but there was no significant age difference amongst NW, overweight, and obese participants. Total plasma cholesterol, LDL, and HDL levels were comparable between people with and without diabetes while triglycerides were significantly higher in diabetic and lower in lean non-diabetic participants, and HDL level was significantly higher in NW non-diabetic participants than in other groups (Table 2).

### 3.2. AT SRA1 Expression in Obesity and Type 2 Diabetes

**Figure 1:**
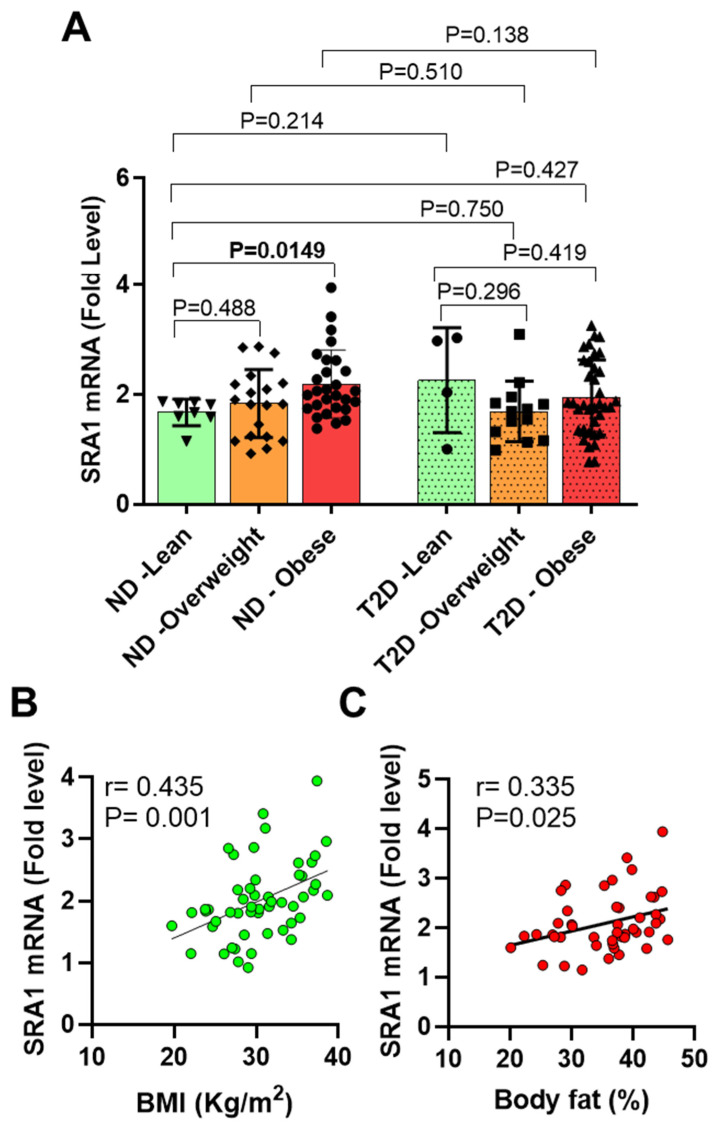
Increased adipose tissue SRA expression in obese individuals without diabetes. Adipose tissue samples were obtained from individuals with various BMI. Samples were divided into lean, overweight, and obese sub-groups. Total cellular RNA was isolated from adipose tissue, and gene expression was determined by real time RT-PCR. Relative mRNA expression was presented as fold SRA1 change. Each dot represents the individual value of SRA1, and the line represents mean value. (**A**) SRA1 expression in lean, overweight, and obese individuals without and with diabetes. ND represents individuals without diabetes. T2D represents individuals with type 2 diabetes; (**B**) correlation between SRA1 gene expression and BMI (kg/m^2^) in ND population; (**C**) correlation between SRA1 gene expression and body fat (%) in ND population. Data are represented as mean ± SEM. Statistical analysis between groups was performed using two-tailed Student’s *t*-test. *p* < 0.05 was considered as statistically significant.

**Figure 2:**
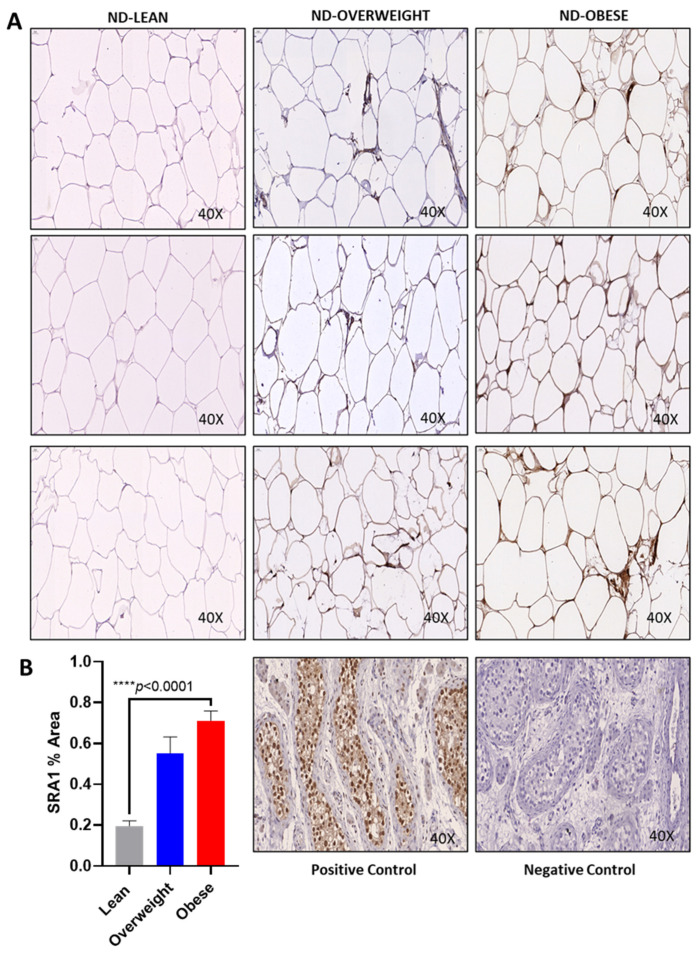
Increased SRA1 protein expression in obese adipose tissue. (**A**,**B**) Increased SRA1 protein expression in obese adipose tissue. Adipose SRA1 protein expression was determined by immunohistochemistry (IHC) in 8 lean, 6 overweight, and 9 obese individuals. The representative images obtained from five independent determinations with similar results show elevated adipose SRA1 protein expression in overweight and obese individuals compared with lean: (**A**) 40× magnification of IHC images. Statistical analysis between groups was performed using two-tailed Student’s *t*-test. *p* < 0.05 was considered as statistically significant.

In humans, the level of SRA1 adipose expression and its relationship with clinical and immunometabolic signatures of obesity and T2D are poorly understood. We therefore asked whether obesity and T2D affected the expression of SRA1 in the adipose tissue. We found that *SRA1* expression was significantly higher (*n* = 28, 2.19 ± 0.62 fold, *p* = 0.015) in non-diabetic obese compared with non-diabetic NW (*n* = 8, 1.67 ± 0.24 fold) participants (Table 2, Figure 1A). In individuals without T2D, immunohistochemistry analysis showed high SRA1 protein expression in obese individuals when compared with NW (lean) (Figure 2A,B). Whereas *SRA1* expression differed non-significantly between diabetic and non-diabetic participants (Table 2, Figure 1B). Additionally, no significant difference was detected between NW (1.87 ± 0.61 fold, *n* = 12), overweight (1.77 ± 0.59 fold, *n* = 32) and obese (2.04 ± 0.66 fold, *n* = 64), diabetic, and non-diabetic participants combined (Figure 1C). No difference in SRA1 protein expression was seen in individuals with diabetes (Supplementary Figure S1).

*SRA1* expression was associated directly with BMI (*r* = 0.221, *p* = 0.022), PBF (*r* = 0.216, *p* = 0.044), fasting serum insulin (*r* = 0.0242, *p* = 0.026), and HOMA-IR (*r* = 0.235, *p* = 0.030) (Table 3). In non-diabetic participants SRA1 expression was associated directly with BMI (*r* = 0.435, *p* = 0.001), PBF (*r* = 0.335, *p* = 0.025) (Figure 1B,C), waist circumference (*r* = 0.291, *p* = 0.047), fasting serum insulin (*r* = 0.440, *p* = 0.005), and HOMA-IR (*r* = 0.433, *p* = 0.005). However, in individuals with T2D, only the HbA1c associated inversely with the levels of *SRA1* expression. Correlational analysis of *SAR1* adipose expression with clinico-metabolic signatures, after stratifying the study population based on BMI regardless of the diabetes status, indicated that *SRA1* expression was associated directly with waist circumference (*r* = 0.0646, *p* = 0.043) in NW, and inversely with HbA1c (*r* = −0.417, *p* = 0.022) in overweight individuals (Table 3).

### 3.3. Association of AT SRA1 Expression with Inflammatory Signatures

**Figure 3:**
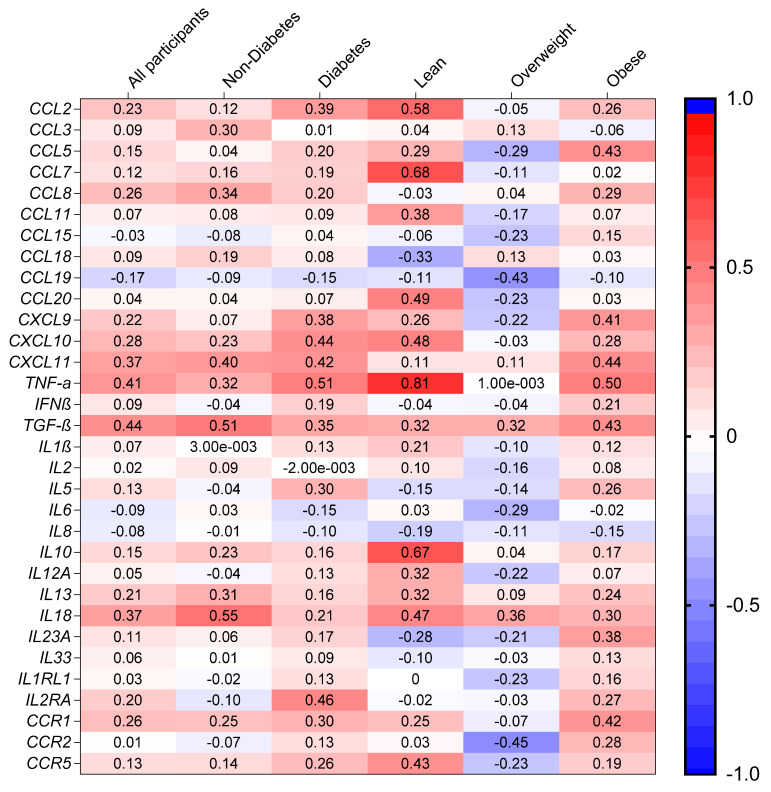
Heat map of the correlation of SRA1 expression with inflammatory markers in adipose tissue.

The most significant change in the adipose tissue in obesity, from the immunological standpoint, is its increased infiltration by monocytes/macrophages and a phenotypic shift of resident macrophages from an anti-inflammatory M2 to an inflammatory M1 type, which explains the persistence of chronic low-grade inflammation in the adipose compartment. Since *SRA1* adipose tissue expression was elevated in obesity, we next sought to determine the relationship of *SRA1* expression with the inflammatory cytokines/chemokines in our study population. AT *SRA1* expression in our total study population associated directly with several inflammatory markers in fat including *CCL2* (*r* = 0.234, *p* = 0.017), *CCL8* (*r* = 0.262, *p* = 0.011), *CXCL9* (*r* = 0.215, *p* = 0.029), *CXCL10* (*r* = 0.280, *p* = 0.004), *CXCL11* (*r* = 0.368, *p* < 0.001), *TNF-α* (*r* = 0.413, *p* < 0.0001), *TGF-β* (*r* = 0.438, *p* < 0.0001), *IL13* (*r* = 0.205, *p* = 0.044), *IL18* (*r* = 0.371, *p* < 0.001), *IL2RA* (*r* = 0.197, *p* = 0.041), and *CCR1* (*r* = 0.258, *p* = 0.009) (Table 4, Heat map shown in Figure 3). In people without diabetes *SRA1* expression was associated with *CCL3* (*r* = 0.298, *p* = 0.036), *CCL8* (*r* = 0.344, *p* = 0.021), *CXCL11* (*r* = 0.400, *p* = 0.003), *TNF-α* (*r* = 0.317, *p* = 0.030), *TGF-β* (*r* = 0.514, *p* < 0.0001), *IL13* (*r* = 0.310, *p* = 0.028), and *IL18* (*r* = 0.547, *p* < 0.0001). In people with T2D *SRA1* expression was associated directly with the transcripts expression of *CCL2* (*r* = 0.385, *p* = 0.006), *CXCL9* (*r* = 0.379, *p* = 0.008), *CXCL10* (*r* = 0.437, *p* = 0.002), *CXCL11* (*r* = 0.421, *p* = 0.002), *TNF-α* (*r* = 0.505, *p* < 0.001), *TGF-β* (*r* = 0.348, *p* = 0.014), *IL5* (*r* = 0.297, *p* = 0.040), *IL2RA* (*r* = 0.461, *p* = 0.001), and *CCR1* (*r* = 0.299, *p* = 0.033). *SRA1* adipose expression in overweight population had a direct association with *CCL7* (*r* = 0.682, *p* = 0.021), *TNF-α* (*r* = 0.810, *p* = 0.015), and *IL10* (*r* = 0.667, *p* = 0.050) and an inverse association with *CCL19* (*r* = −0.427, *p* = 0.017), and *CCR2* (*r* = −0.453, *p* = 0.009). In obese participants *SRA1* expression was associated with a wide spectrum of inflammatory markers including *CCL2* (*r* = 0.256, *p* = 0.043), *CCL5* (*r* = 0.433, *p* = 0.002), *CCL8* (*r* = 0.294, *p* = 0.027), *CXCL9* (*r* = 0.408, *p* = 0.001), *CXCL10* (*r* = 0.282, *p* = 0.028), *CXCL11* (*r* = 0.440, *p* < 0.001), *TNF-α* (*r* = 0.504, *p* < 0.0001), *TGF-β* (*r* = 0.430, *p* = 0.001), *IL5* (*r* = 0.264, *p* = 0.039), *IL18* (*r* = 0.295, *p* = 0.021), *IL23A* (*r* = 0.379, *p* = 0.002), *IL2RA* (*r* = 0.274, *p* = 0.028), *CCR1* (*r* = 0.417, *p* = 0.001), and *CCR2* (*r* = 0.278, *p* = 0.046) (Table 4; Heat map shown in Figure 3).

We observed that inflammatory markers (TNF-α and IL-18) protein expression was positively correlated with SRA1 protein expression in individuals without diabetes (Supplementary Figure S2). We also observed inflammatory markers (IL-8, TNF-α and CCL2) protein expression was positively correlated with SRA1 protein expression in individuals with diabetes (Supplementary Figure S3).

### 3.4. Analysis of the Indipenedent Associations between SRA1 and Immune Metabolic Markers

In order to determine independent associations of *SRA1* with immune metabolic markers, the markers having significant associations with *SRA1* expression level were further analyzed by multivariable stepwise linear regression analysis (Table 5). In the total population (*n* = 108) *IL-18, TGF-β*, and *CXCL11* were independently associated with *SRA1* expression. *TGF-β* and *IL18* in people without diabetes and *TNF-α* and *IL2RA* in people with diabetes were independent predictors of *SRA1* expression. Regarding the obesity status, independent associations were found only for *TNF-α* and *CCR2* with *SRA1* expression in NW (*n* = 12), and overweight (*n* = 32) participants. In obese participants (*n* = 64), both *TNF-α* and *IL18* were detected as independent predictors for *SRA1* expression (Table 5).

## 4. Discussion

Adipose tissues function both as reservoirs of fuel and as endocrine cells, secreting adipokines, chemokines, and cytokines to regulate energy metabolism and glucose homeostasis [26,27]. Obesity induces a state of chronic, low-grade inflammation in the adipose tissue that is accompanied by the local secretion of several pro-inflammatory cytokines and chemokines, especially TNFα, attenuating insulin action and resulting in insulin resistance through activation of the JNK pathway [20,34]. Persistence of a low-grade inflammatory state from obesity, over time, leads to the onset of well-known obesity-associated inflammatory diseases including T2D, cardiovascular disease, atherosclerosis, hepatic steatosis, hypertension, sarcopenia, osteoarthritis, rheumatoid arthritis, and cancer.

In mammals, including humans, *SRA1* gene expresses a long non-coding steroid receptor RNA activator 1 (SRA1) in high energy demand tissues such as adipose tissue, liver, heart, and muscle [1,2,3]. Identified in 1999 using a yeast two-hybrid assay for human B-cell library and the activation function one domain of progesterone receptor as bait, SRA1 is known to affect or regulate a wide variety of physiological and pathological processes including hepatic steatosis, mammary gland development, steroidogenesis, tumorigenesis, stem cell function, myocyte, and adipocyte differentiation [35]. It was shown that SRA1 is expressed at the highest levels in the adipocyte fraction of white adipose tissue, followed by brown adipose tissue, and the preadipocytes [36,37]. SRA1 has been shown to act as an RNA coactivator of nuclear receptors, involved in the regulation of adipocyte differentiation, and in glucose homeostasis and insulin sensitivity in adipocytes [5,8,13].

Based on our knowledge, this is the first study investigating the level of expression of *SRA1* in the AT in humans with obesity/T2D. In this study, we found that adipose *SRA1* expression was significantly higher in obese compared to lean non-diabetic participants. Nonetheless, no significant differences were detected between diabetic vs. non-diabetic participants, as well as among NW, overweight, and obese participants with diabetes. Regarding the association of *SRA1* expression with metabolic markers, BMI, PBF, serum insulin, and HOMA-IR levels associated directly with *SRA1* expression in the total study population as well as in non-diabetic participants. Waist circumference associated directly with *SRA1* expression in non-diabetic and lean participants whereas HbA1c associated inversely with *SRA1* expression in overweight participants. Similarly, Liu et al. found that expression of lncRNA GYG2P1 and Sun et al. found that expression of lncRNA p21015 were inversely associated with adiposity parameters including BMI, waist circumference, fasting serum insulin, and triglycerides [38,39]. Overall, our data showed varying association patterns of adipose *SRA1* expression with the metabolic profile of individuals, differing with regard to the obesity and T2D status.

We further extended our analysis to assess the associations between adipose tissue *SRA1* expression and local expression of a wide range of proinflammatory cytokines/chemokines and other immune markers. To this end, in most study populations, adipose *SRA1* expression was directly associated with that *of CXCL9, CXCL10, CXCL11, TNF-α, TGF-β, IL2RA*, and *IL18*. Interestingly, we did not find any association between SRA1 and IL6 adipose expression in this study. *SRA1* expression was associated inversely with that of *CCL19* and *CCR2*.These findings suggest that *SRA1* expression is associated, for the most part, with the expression of inflammatory immune markers in the fat. Similarly, several groups found that the expression of lncRNAs such as ANRIL and ASMER1/2 correlated directly with the proinflammatory factors and inflammatory pathways [40,41]. Gao et al. identified 86 differentially expressed lncRNAs between obese and non-obese persons while 44 lncRNAs were differentially expressed in individuals who were insulin-resistant versus insulin-sensitive and obese people [41].

Besides, TGF-β and IL18 independently predicted the *SRA1* expression in non-diabetics as well as in the total (diabetic and non-diabetic) study population, while *TNF-α* and *IL-2RA* were the independent predictors of *SRA1* only in people with diabetes. TNF-α also predicted *SRA1* adipose expression in both NW and obese populations, regardless of diabetes status. These data revealed specific association patterns of adipose *SRA1* expression with typical immune markers, mostly inflammatory by nature.

To investigate the functional role of SRA, a SRA knockout mouse model was developed [36]. Body composition analysis of this mouse model revealed significant differences in reduced total body weight, percentage of fat mass, epididymal white fat mass, subcutaneous white fat mass, and liver mass, with an increased percentage of lean mass [36]. The reduced fat mass was associated with small adipocytes compared with wild-type counterparts [36]. Interestingly, the SRA knockout mouse model displayed an improved insulin sensitivity and resistance to developing obesity in high-fat diet conditions, reduced fatty liver, and improved glucose tolerance [36,37]. The improvement of insulin sensitivity was associated with reduced inflammatory signaling, including reduced plasma TNFα levels and with a reduced expression of inflammation genes including *TNF-α* and *CCl2* in white adipose tissue, but an unchanged level of IL6 [36,37]. In addition, microarray data obtained from an adipocyte cell line revealed several SRA-responsive genes including genes related to cell cycle, serum insulin, and TNF-α signaling pathways [37]. These results suggest an important role of SRA in adipose tissue development and function, providing a potential target to control obesity and metabolic syndrome. It is important to keep in consideration that the SRA knockout mouse model has a global loss of SRA expression in all tissues including liver and fat tissue, which may contribute to whole-body insulin sensitivity. Therefore, further studies using both gain of function and loss of function (tissue-specific knockout) models, targeting SRA expression exclusively in the liver, fat, or muscle will be indispensable to elucidate the role of SRA in regulating body metabolism and glucose homeostasis, as a potential target to control obesity and T2D. In a knock-out mouse model of SRA1 has displayed improved insulin sensitivity and resistance to developing obesity under high-fat diet conditions, but obesity and T2D themselves might have a non-significant influence on SRA1 expression levels, reflecting a plausible absence of reverse causation.

Our results support the opinion that adipose expression of SRA1 may be viewed as a potential new biomarker of obesity in humans, but caution should be considered in generalizing these findings as our study is limited by certain caveats. First, it is a cross-sectional study comprising a limited number of participants stratified by their obesity and diabetes status. Second, it is a clinical study and the results are exclusively based on a correlational analysis of the *SRA1* expression versus immune–metabolic markers gene expression in the subcutaneous fat tissue. Therefore, the obesity/T2D-related changes in the visceral fat compartment which are more representative and indicative of the pathophysiological effects of obesity or diabetes remain unclear. Third, our data also lack information of the *SRA1* and SRA protein (SRAP) expression in main insulin target tissues other than white subcutaneous adipose tissue in obesity. Indeed, the functions and mechanisms of action of most of the lncRNAs remain elusive thus far, while only a small number of lncRNAs have been well characterized to provide the mechanistic insights by which lncRNAs exert their diverse effects/functions. A substantial argument in favor of the pathophysiological significance of SRA1 as a regulatory molecular signal in obesity lies in its variable expression that we observed between non-diabetic NW and obese people, implying that further clinical studies, preferably longitudinal, and including larger cohorts as well as experimental studies of loss and gain of function approaches will be needed to understand the SRA1-associated underlying molecular mechanisms at the genetic and epigenetic levels that regulate metabolic disease pathogenesis.

## 5. Conclusion

In conclusion, we show for the first time that SRA1 adipose expression is elevated with obesity in humans, which correlates with specific metabolic parameters and/or adipose tissue immune markers. A potential link between elevated SRA1 expression and typical correlates of obesity/T2D implies that SRA1 may have significance as a potential new biomarker of metabolic disorders.

## Figures and Tables

**Table 1:** List of TaqMan gene expression assays.

Gene Name	Assay ID	Gene Name	Assay ID
*SRA1*	Hs00398296_g1	*IL1β*	Hs01555410_m1
*CCL2*	Hs00234140_m1	*IL2*	Hs00174114_m1
*CCL3*	Hs04194942_s1	*IL5*	Hs01548712_g1
*CCL5*	Hs00982282_m1	*IL6*	Hs00985639_m1
*CCL7*	Hs00171147_m1	*IL8*	Hs00174103_m1
*CCL8*	Hs04187715_m1	*IL10*	Hs00961622_m1
*CCL11*	Hs00237013_m1	*IL12A*	Hs01073447_m1
*CCL15*	Hs00361122_m1	*IL13*	Hs00174379_m1
*CCL18*	Hs00268113_m1	*IL18*	Hs01038788_m1
*CCL19*	Hs00171149_m1	*IL23A*	Hs00900828_g1
*CCL20*	Hs01011368_m1	*IL33*	Hs00369211_m1
*CXCL9*	Hs00171065_m1	*IL1RL1*	Hs00545033_m1
*CXCL10*	Hs01124251_g1	*IL2RA*	Hs00907779_m1
*CXCL11*	Hs04187682_g1	*CCR1*	Hs00928897_s1
*TNF-α*	Hs01113624_g1	*CCR2*	Hs00704702_s1
*IFNβ*	Hs01077958_s1	*CCR5*	Hs99999149_s1
*TGF-β*	Hs00820148_g1	*GAPDH*	Hs03929097_g1

**Table 2:** Demographic and clinical characteristics of study population.

	Total Participants (*n* = 108)										
	Non-Diabetic (*n* = 55)					Diabetic (*n* = 53)					
	Lean	Overweight	Obese	Lean vs. Overweight	Lean vs. Obese	Lean	Overweight	Obese	Lean vs. Overweight	Lean vs. Obese	Non-Diabetic vs. Diabetic
	(*n* = 8)	(*n* = 19)	(*n* = 28)			(*n* = 4)	(*n* = 13)	(*n* = 36)			
				(*p*)	(*p*)				(*p*)	(*p*)	(*p*)
Age (years)	42.00 (36.00–50.25)	42.00 (35.00–52.00)	46.50 (36.00–59.00)	0.688	0.387	56 (49.75–57.75)	54.00 (53.00–57.00)	53.00 (46.00–58.00)	0.608	0.557	<0.001
Weight (kg)	61.00 (58.40–77.33)	79.00 (70.20–88.00)	91.75 (84.83–105.70)	0.004	<0.0001	68.85 (57.9–69.75)	77.7 (72.5–85.25)	93.50 (83.08–99.70)	0.024	0.001	0.327
Height (cm)	1.66 (1.55–1.80)	1.67 (1.59–1.76)	1.65 (1.55–1.73)	0.906	0.422	1.65 (1.538–1.665)	1.66 (1.61–1.72)	1.69 (1.58–1.73)	0.461	0.223	0.784
BMI (kg/m^2^)	23.81 (22.07–24.56)	28.43 (27.26–29.4)	35.20 (31.65–37.23)	<0.0001	<0.0001	24.99 (24.28–25.59)	28.2 (27.13–28.87)	33.35 (31.47–35.56)	0.013	0.001	0.417
Waist circumference (cm)	81.00 (77.00–97.00)	96 (89–101.8)	109.00 (99.00–117.00)	0.014	<0.0001	82 (76–87)	98.00 (93.00–103.00)	110 (104.5–114)	0.024	0.005	0.052
HIP circumference (cm)	103.00 (91.50–105.00)	103.50 (97.00–111.5)	120.00 (109.50–125.00)	0.324	<0.0001	100.00 (93.00–106.00)	104.00 (101.00–111.00)	113.80 (110.00–120.80)	0.273	0.015	0.768
WHR	0.82 (0.74–0.94)	0.95 (0.82–1.02)	0.91 (0.81–1.00)	0.105	0.072	0.8172 (0.78–0.87)	0.93 (0.90–1.01)	0.9571 (0.8789–1.018)	0.016	0.021	0.089
Body fat (%)	26.90 (22.30–36.70)	30.9 (28.43–37.63)	39.90 (36.78–43.63)	0.074	<0.0001	34.8 (27.70–36.50)	30.7 (26.00–37.30)	38 (32.2–41.85)	0.938	0.181	0.984
Fasting plasma glucose (mM)	4.90 (4.32–5.18)	5.1 (4.9–5.43)	5.20 (4.75–5.88)	0.27	0.186	7.7 (5.65–9.953)	8.72 (6.92–11.13)	8.15 (6.6–10.03)	0.362	0.471	<0.0001
Triglycerides (mmol/L)	0.62 (0.43–0.89)	1.3 (0.64–1.65)	1.05 (0.72–1.53)	0.014	0.042	0.97 (0.47–1.88)	1.79 (0.84–2.40)	1.395 (1.113–1.818)	0.225	0.131	<0.001
Total cholesterol (mmol/L)	5.35 (3.88–6.04)	5 (4.48–5.42)	4.75 (4.16–6.05)	0.769	0.943	4.48 (3.8–6.54)	4.09 (3.85–5.56)	4.94 (4.1–5.745)	0.544	0.701	0.314
HDL cholesterol (mmol/L)	1.59 (1.18–2.01)	1.2 (1.08–1.36)	1.16 (1.02–1.38)	0.033	0.041	1.14 (0.97–1.3)	1.03 (0.89–1.31)	1.11 (0.95–1.36)	0.671	0.835	0.096
LDL (mmol/L)	3.55 (2.33–3.93)	3.1 (2.6–3.7)	3.25 (2.60–4.00)	0.995	0.746	2.9 (2.38–4.63)	2.3 (1.8–3.55)	2.85 (2.275–3.75)	0.202	0.686	0.069
HbA1c (%)	5.50 (5.15–5.85)	5.5 (5.18–5.73)	5.83 (5.43–5.98)	0.946	0.404	7.3 (5.90–8.70)	7.7 (6.575–10.18)	8.1 (7.35–9.175)	0.544	0.367	<0.0001
Fasting insulin (mU/L)	5.56 (5.04–11.51)	6.08 (4.99–15.67)	17.38 (7.32–34.58)	0.448	0.015	13.37 (4.08–30.63)	10.37 (6.277–17.16)	17.15 (7.909–43.42)	0.735	0.48	0.008
HOMA-IR	1.31 (1.00–2.53)	1.351 (1.16–3.61)	4.09 (1.89–10.91)	0.419	0.01	5.53 (1.11–7.49)	3.03 (2.59–6.01)	5.74 (3.078–17.69)	0.735	0.377	<0.0001
*SRA1* mRNA(Fold level)	1.74 (1.59–1.85)	1.822 (1.23–2.20)	2.03 (1.75–2.57)	0.489	0.015	2.51 (1.26–3.01)	1.69 (1.24–1.90)	1.814 (1.35–2.567)	0.296	0.419	0.373

BMI, body mass index; WHR, Waist/Hip ratio; HDL, high-density lipoprotein; LDL, low-density lipoprotein; HbA1c, glycated hemoglobin.

**Table 3:** Correlation of *SRA1* expression level with various clinical and biochemical markers.

	All Participants (*n* = 108)		Non-Diabetic (*n* = 55)		Diabetic (*n* = 53)		Lean (*n* = 12)		Overweight (*n* = 32)		Obese (*n* = 64)	
	*r*	*p*	*r*	*p*	*r*	*p*	*r*	*p*	*r*	*p*	*r*	*p*
Age	0.052	0.594	0.102	0.458	0.075	0.596	0.351	0.263	0.047	0.800	−0.007	0.957
Weight	0.092	0.344	0.200	0.143	0.012	0.930	0.664 *	0.018	0.154	0.399	−0.136	0.285
Height	−0.109	0.263	−0.144	0.294	−0.082	0.562	0.423	0.170	0.094	0.608	−0.280 *	0.025
BMI	0.221 *	0.022	0.435 **	0.001	0.121	0.39	0.552	0.063	0.192	0.294	0.125	0.327
PBF	0.216 *	0.044	0.335 *	0.025	0.027	0.863	−0.248	0.489	−0.095	0.639	0.214	0.128
Waist	0.121	0.26	0.291 *	0.047	0.02	0.902	0.646 *	0.043	0.077	0.698	−0.084	0.559
Hip	0.156	0.141	0.157	0.287	0.15	0.343	0.334	0.345	0.176	0.371	−0.026	0.855
WHR	−0.004	0.974	0.163	0.274	−0.081	0.612	0.309	0.385	−0.053	0.791	−0.036	0.804
GLU	−0.025	0.797	0.075	0.586	−0.088	0.535	0.249	0.436	−0.138	0.46	−0.058	0.646
TGL	0.007	0.94	0.200	0.143	−0.097	0.495	0.063	0.846	0.075	0.690	−0.076	0.548
Chol	0.004	0.971	0.023	0.866	−0.043	0.762	0.042	0.897	0.028	0.882	0.005	0.967
HDL	0.073	0.459	−0.015	0.913	0.164	0.249	−0.427	0.167	0.082	0.661	0.183	0.151
LDL	−0.017	0.863	−0.042	0.760	−0.006	0.965	0.074	0.819	−0.093	0.619	0.014	0.914
HbA1c	−0.163	0.096	0.164	0.235	−0.349 *	0.011	0.399	0.198	−0.417 *	0.022	−0.214	0.089
Insulin	0.242 *	0.026	0.44 **	0.005	0.065	0.673	0.009	0.979	0.277	0.162	0.179	0.229
HOMA-IR	0.235 *	0.03	0.433 **	0.005	0.108	0.481	0.309	0.355	0.192	0.337	0.174	0.242

BMI, body mass index; PBF, percent body fat; WHR, waist/hip ratio; GLU, fasting plasma glucose; TGL, plasma triglycerides; Chol, cholesterol; HDL, high-density lipoprotein; LDL, low-density lipoprotein; HbA1c, glycated hemoglobin; HOMA-IR, homeostatic model assessment. * *p* < 0.05, and ** *p* < 0.01.

**Table 4:** Correlation of the *SRA1* expression levels with inflammatory markers in adipose tissue.

	All Participants (*n* = 108)		Non-Diabetic (*n* = 55)		Diabetic (*n* = 53)		Lean (*n* = 12)		Overweight (*n* = 32)		Obese (*n* = 64)	
	*r*	*p*	*r*	*p*	*r*	*p*	*r*	*p*	*r*	*p*	*r*	*p*
*CCL2*	0.234 *	0.017	0.122	0.384	0.385 **	0.006	0.583	0.099	−0.045	0.809	0.256 *	0.043
*CCL3*	0.091	0.366	0.298 *	0.036	0.011	0.938	0.036	0.915	0.132	0.470	−0.057	0.669
*CCL5*	0.146	0.170	0.037	0.810	0.201	0.180	0.286	0.493	−0.291	0.106	0.433 **	0.002
*CCL7*	0.120	0.229	0.161	0.260	0.187	0.190	0.682 *	0.021	−0.106	0.583	0.019	0.882
*CCL8*	0.262 *	0.011	0.344 *	0.021	0.202	0.169	−0.033	0.932	0.041	0.839	0.294 *	0.027
*CCL11*	0.065	0.517	0.075	0.603	0.093	0.511	0.382	0.276	−0.165	0.375	0.070	0.594
*CCL15*	−0.033	0.739	−0.078	0.573	0.036	0.799	−0.056	0.863	−0.234	0.198	0.148	0.246
*CCL18*	0.092	0.353	0.188	0.178	0.075	0.600	−0.333	0.347	0.132	0.472	0.028	0.827
*CCL19*	−0.165	0.098	−0.085	0.550	−0.145	0.315	−0.112	0.729	−0.427 *	0.017	−0.101	0.446
*CCL20*	0.037	0.711	0.039	0.783	0.066	0.643	0.491	0.150	−0.233	0.208	0.034	0.791
*CXCL9*	0.215 *	0.029	0.069	0.618	0.379 **	0.008	0.264	0.433	−0.215	0.245	0.408 **	0.001
*CXCL10*	0.280 **	0.004	0.232	0.092	0.437 **	0.002	0.479	0.162	−0.028	0.882	0.282 *	0.028
*CXCL11*	0.368 ***	<0.001	0.400 **	0.003	0.421 **	0.002	0.112	0.729	0.111	0.561	0.440 ***	<0.001
*TNF-α*	0.413 ****	<0.0001	0.317^*^	0.030	0.505 ***	<0.001	0.810^*^	0.015	0.001	0.995	0.504 ****	<0.0001
*IFNβ*	0.094	0.336	−0.036	0.798	0.189	0.175	−0.035	0.914	−0.044	0.814	0.209	0.100
*TGF-β*	0.438 ****	<0.0001	0.514 ****	<0.0001	0.348 *	0.014	0.321	0.365	0.317	0.082	0.430 **	0.001
*IL1β*	0.068	0.547	0.003	0.983	0.127	0.436	0.214	0.645	−0.102	0.622	0.119	0.422
*IL2*	0.021	0.834	0.093	0.510	−0.002	0.991	0.098	0.762	−0.160	0.390	0.077	0.550
*IL5*	0.132	0.191	−0.043	0.764	0.297 *	0.040	−0.145	0.670	−0.141	0.483	0.264 *	0.039
*IL6*	−0.089	0.378	0.033	0.820	−0.145	0.309	0.033	0.932	−0.294	0.115	−0.023	0.861
*IL8*	−0.075	0.465	−0.010	0.948	−0.100	0.484	−0.190	0.651	−0.108	0.561	−0.148	0.269
*IL10*	0.152	0.129	0.226	0.107	0.163	0.264	0.667 *	0.050	0.043	0.814	0.173	0.186
*IL12A*	0.051	0.641	−0.036	0.822	0.126	0.404	0.321	0.482	−0.216	0.270	0.070	0.621
*IL13*	0.205 *	0.044	0.310 *	0.028	0.158	0.288	0.321	0.365	0.091	0.627	0.236	0.080
*IL18*	0.371 ***	<0.001	0.547 ****	<0.0001	0.214	0.144	0.467	0.205	0.356	0.058	0.295 *	0.021
*IL23A*	0.112	0.252	0.056	0.688	0.169	0.227	−0.280	0.379	−0.205	0.259	0.379 **	0.002
*IL33*	0.056	0.572	0.011	0.940	0.087	0.534	−0.098	0.762	−0.026	0.888	0.134	0.298
*IL1RL1*	0.034	0.748	−0.024	0.872	0.129	0.382	0.000	1.000	−0.231	0.219	0.163	0.226
*IL2RA*	0.197 *	0.041	−0.098	0.478	0.461 **	0.001	−0.021	0.948	−0.033	0.859	0.274 *	0.028
*CCR1*	0.258 **	0.009	0.246	0.082	0.299 *	0.033	0.248	0.489	−0.067	0.717	0.417 **	0.001
*CCR2*	0.005	0.962	−0.066	0.660	0.132	0.400	0.029	0.957	−0.453 **	0.009	0.278 *	0.046
*CCR5*	0.134	0.176	0.144	0.301	0.260	0.071	0.430	0.214	−0.227	0.227	0.194	0.129

CCL, Chemokine (C-C motif) ligand; CXCL, chemokine (C-X-C motif) ligand; TNF-α, tumor necrosis factor alpha; IFNβ,Interferon beta; TGF-β, transforming growth factor beta; IL, interleukin;. CCR, C-C chemokine receptor. * *p* < 0.05, ** *p* < 0.01, *** *p* < 0.001, and **** *p* < 0.0001.

**Table 5:** Multiple linear regression analysis.

Multiple Regression Analysis				
All participants (*n* = 108)	ANOVA	R^2^ = 0.29	*p* value < 0.0001	
	Predictor Variable	*TGF-β*	β value = 0.287	*p* value = 0.004
		*IL18*	β value = 0.286	*p* value = 0.004
		*CXCL11*	β value = 0.237	*p* value = 0.009
Non-Diabetic (*n* = 55)	ANOVA	R^2^ = 0.41	*p* value < 0.0001	
	Predictor Variable	*TGF-β*	β value = 0.419	*p* value = 0.002
		*IL18*	β value = 0.326	*p* value = 0.014
Diabetic (*n* = 53)	ANOVA	R^2^ = 0.24	*p* value = 0.0001	
	Predictor Variable	*TNF-α*	β value = 0.434	*p* value = 0.001
		*IL2RA*	β value = 0.259	*p* value = 0.044
Lean (*n* = 12)	ANOVA	R^2^ = 0.79	*p* value = 0.002	
	Predictor Variable	*TNF-α*	β value = 0.90	*p* value = 0.002
Overweight (*n* = 32)	ANOVA	R^2^ = 0.158	*p* value = 0.014	
	Predictor Variable	*CCR2*	β value = −0.430	*p* value = 0.014
Obese (*n* = 64)	ANOVA	R^2^ = 0.33	*p* value < 0.0001	
	Predictor Variable	*TNF-α*	β value = 0.412	*p* value = 0.001
		*IL18*	β value = 0.311	*p* value = 0.010

## Data Availability

The data presented in this study are available on request from the corresponding author.

## Supplementary Materials

The following are available online at https://www.mdpi.com/article/10.3390/cells10102602/s1, Figure S1: SRA1 protein expression in the adipose tissue of the individuals with diabetes. Figure S2: Increased TNF-α and IL-18 correlated with SRA1 protein expression in adipose tissue. Figure S3: IL-8, TNF-α and CCL2 protein expression in correlated with SRA1 protein expression in adipose tissue. Table S1: Correlation of SRA1 expression level with various clinical and biochemical markers.

## References

[1] Lanz R.B., McKenna N.J., Onate S.A., Albrecht U., Wong J., Tsai S.Y., Tsai M.-J., O’Malley B.W. (1999). A steroid receptor coactivator, sra, functions as an rna and is present in an src-1 complex. Cell.

[2] Emberley E., Huang G.-J., Hamedani M.K., Czosnek A., Ali D., Grolla A., Lu B., Watson P.H., Murphy L.C., Leygue E. (2003). Identification of new human coding steroid receptor rna activator isoforms. Biochem. Biophys. Res. Commun..

[3] Friedrichs F., Zugck C., Rauch G.J., Ivandic B., Weichenhan D., Müller-Bardorff M., Meder B., Mokhtari N.E.E., Regitz-Zagrosek V., Hetzer R. (2009). Hbegf, sra1, and ik: Three cosegregating genes as determinants of cardiomyopathy. Genome Res..

[4] Hubé F., Velasco G., Rollin J., Furling D., Francastel C. (2011). Steroid receptor rna activator protein binds to and counteracts sra rna-mediated activation of myod and muscle differentiation. Nucleic Acids Res..

[5] Colley S.M., Leedman P.J. (2011). Steroid receptor rna activator—A nuclear receptor coregulator with multiple partners: Insights and challenges. Biochimie.

[6] Beato M., Vicent G.P. (2013). A new role for an old player: Steroid receptor rna activator (sra) represses hormone inducible genes. Transcription.

[7] Leygue E. (2007). Steroid receptor rna activator (sra1): Unusual bifaceted gene products with suspected relevance to breast cancer. Nucl. Recept. Signal..

[8] Kotan L.D., Cooper C., Darcan Ş., Carr I.M., Özen S., Yan Y., Hamedani M.K., Gürbüz F., Mengen E., Turan İ. (2016). Idiopathic hypogonadotropic hypogonadism caused by inactivating mutations in sra1. J. Clin. Res. Pediatr. Endocrino..

[9] Lin K., Ma J., Wu R., Zhou C., Lin J. (2014). Influence of ovarian endometrioma on expression of steroid receptor rna activator, estrogen receptors, vascular endothelial growth factor, and thrombospondin 1 in the surrounding ovarian tissues. Reprod. Sci..

[10] Liu Z., Hao C., Huang X., Zhang N., Bao H., Qu Q. (2015). Peripheral blood leukocyte expression level of lncrna steroid receptor rna activator (sra) and its association with polycystic ovary syndrome: A case control study. Gynecol. Endocrinol..

[11] Caretti G., Schiltz R.L., Dilworth F.J., Di Padova M., Zhao P., Ogryzko V., Fuller-Pace F.V., Hoffman E.P., Tapscott S.J., Sartorelli V. (2006). The rna helicases p68/p72 and the noncoding rna sra are coregulators of myod and skeletal muscle differentiation. Dev. Cell.

[12] Xu B., Yang W.-H., Gerin I., Hu C.-D., Hammer G.D., Koenig R.J. (2009). Dax-1 and steroid receptor rna activator (sra) function as transcriptional coactivators for steroidogenic factor 1 in steroidogenesis. J. Mol. Cell Biol.

[13] Murphy L.C., Simon S.L., Parkes A., Leygue E., Dotzlaw H., Snell L., Troup S., Adeyinka A., Watson P.H. (2000). Altered expression of estrogen receptor coregulators during human breast tumorigenesis. Cancer Res..

[14] Cooper C., Guo J., Yan Y., Chooniedass-Kothari S., Hube F., Hamedani M.K., Murphy L.C., Myal Y., Leygue E. (2009). Increasing the relative expression of endogenous non-coding steroid receptor rna activator (sra) in human breast cancer cells using modified oligonucleotides. Nucleic Acids Res..

[15] Kurisu T., Tanaka T., Ishii J., Matsumura K., Sugimura K., Nakatani T., Kawashima H. (2006). Expression and function of human steroid receptor rna activator in prostate cancer cells: Role of endogenous hsra protein in androgen receptor-mediated transcription. Prostate Cancer Prostatic Dis..

[16] Lanz R.B., Razani B., Goldberg A.D., O’Malley B.W. (2002). Distinct rna motifs are important for coactivation of steroid hormone receptors by steroid receptor rna activator (sra). Proc. Natl. Acad. Sci. USA.

[17] Leygue E., Dotzlaw H., Watson P.H., Murphy L.C. (1999). Expression of the steroid receptor rna activator in human breast tumors. Cancer Res..

[18] Gesta S., Tseng Y.H., Kahn C.R. (2007). Developmental origin of fat: Tracking obesity to its source. Cell.

[19] Doria A., Patti M.E., Kahn C.R. (2008). The emerging genetic architecture of type 2 diabetes. Cell Metab..

[20] Olefsky J.M., Glass C.K. (2010). Macrophages, inflammation, and insulin resistance. Annu. Rev. Physiol.

[21] Kawai T., Autieri M.V., Scalia R. (2021). Adipose tissue inflammation and metabolic dysfunction in obesity. Am. J. Physiol. Cell Physiol..

[22] Rosa Neto J.C., Calder P.C., Curi R., Newsholme P., Sethi J.K., Silveira L.S. (2021). The immunometabolic roles of various fatty acids in macrophages and lymphocytes. Int. J. Mol. Sci..

[23] Feijóo-Bandín S., Aragón-Herrera A., Moraña-Fernández S., Anido-Varela L., Tarazón E., Roselló-Lletí E., Portolés M., Moscoso I., Gualillo O., González-Juanatey J.R. (2020). Adipokines and inflammation: Focus on cardiovascular diseases. Int. J. Mol. Sci..

[24] Abdelaal M., le Roux C.W., Docherty N.G. (2017). Morbidity and mortality associated with obesity. Ann. Transl. Med..

[25] Phelan S.M., Burgess D.J., Yeazel M.W., Hellerstedt W.L., Griffin J.M., van Ryn M. (2015). Impact of weight bias and stigma on quality of care and outcomes for patients with obesity. Obes. Rev..

[26] Rosen E.D., Spiegelman B.M. (2006). Adipocytes as regulators of energy balance and glucose homeostasis. Nature.

[27] Arner P. (2005). Resistin: Yet another adipokine tells us that men are not mice. Diabetologia.

[28] Sindhu S., Thomas R., Kochumon S., Wilson A., Abu-Farha M., Bennakhi A., Al-Mulla F., Ahmad R. (2019). Increased adipose tissue expression of interferon regulatory factor (irf)-5 in obesity: Association with metabolic inflammation. Cells.

[29] Hill N.R., Levy J.C., Matthews D.R. (2013). Expansion of the homeostasis model assessment of β-cell function and insulin resistance to enable clinical trial outcome modeling through the interactive adjustment of physiology and treatment effects: Ihoma2. Diabetes Care.

[30] Kochumon S., Al-Rashed F., Abu-Farha M., Devarajan S., Tuomilehto J., Ahmad R. (2019). Adipose tissue expression of ccl19 chemokine is positively associated with insulin resistance. Diabetes Metab. Res. Rev..

[31] Ahmad R., Shihab P.K., Thomas R., Alghanim M., Hasan A., Sindhu S., Behbehani K. (2015). Increased expression of the interleukin-1 receptor-associated kinase (irak)-1 is associated with adipose tissue inflammatory state in obesity. Diabetol. Metab. Syndr..

[32] Khadir A., Tiss A., Abubaker J., Abu-Farha M., Al-Khairi I., Cherian P., John J., Kavalakatt S., Warsame S., Al-Madhoun A. (2015). Map kinase phosphatase dusp1 is overexpressed in obese humans and modulated by physical exercise. Am. J. Physiol. Endocrinol. Metab..

[33] Ahmad R., Al-Mass A., Al-Ghawas D., Shareif N., Zghoul N., Melhem M., Hasan A., Al-Ghimlas F., Dermime S., Behbehani K. (2013). Interaction of osteopontin with il-18 in obese individuals: Implications for insulin resistance. PLoS ONE.

[34] Lumeng C.N., Saltiel A.R. (2011). Inflammatory links between obesity and metabolic disease. J. Clin. Investig..

[35] Sheng L., Ye L., Zhang D., Cawthorn W.P., Xu B. (2018). New insights into the long non-coding rna sra: Physiological functions and mechanisms of action. Front. Med..

[36] Liu S., Sheng L., Miao H., Saunders T.L., MacDougald O.A., Koenig R.J., Xu B. (2014). Sra gene knockout protects against diet-induced obesity and improves glucose tolerance*. J. Biol. Chem..

[37] Xu B., Gerin I., Miao H., Vu-Phan D., Johnson C.N., Xu R., Chen X.W., Cawthorn W.P., MacDougald O.A., Koenig R.J. (2010). Multiple roles for the non-coding rna sra in regulation of adipogenesis and insulin sensitivity. PLoS ONE.

[38] Liu Y., Ji Y., Li M., Wang M., Yi X., Yin C., Wang S., Zhang M., Zhao Z., Xiao Y. (2018). Integrated analysis of long noncoding rna and mrna expression profile in children with obesity by microarray analysis. Sci. Rep..

[39] Sun J., Ruan Y., Wang M., Chen R., Yu N., Sun L., Liu T., Chen H. (2016). Differentially expressed circulating lncrnas and mrna identified by microarray analysis in obese patients. Sci. Rep..

[40] Zhou X., Han X., Wittfeldt A., Sun J., Liu C., Wang X., Gan L.-M., Cao H., Liang Z. (2016). Long non-coding rna anril regulates inflammatory responses as a novel component of nf-κb pathway. RNA Biol..

[41] Gao H., Kerr A., Jiao H., Hon C.C., Rydén M., Dahlman I., Arner P. (2018). Long non-coding rnas associated with metabolic traits in human white adipose tissue. EBioMedicine.

