# Supplementary material for: Adipose Tissue Steroid Receptor RNA Activator 1 (SRA1) Expression Is Associated with Obesity, Insulin Resistance, and Inflammation
Source: Cells. 2021 Sep 30;10(10):2602. doi: 10.3390/cells10102602 (PMC8534244; doi:10.3390/cells10102602)
Supplement: Supplementary file 1 [file cells-10-02602-s001.zip › cells-1355059-supplementary.pdf]

Figure S1. SRA1 protein expression in the adipose tissue of the individuals with diabetes

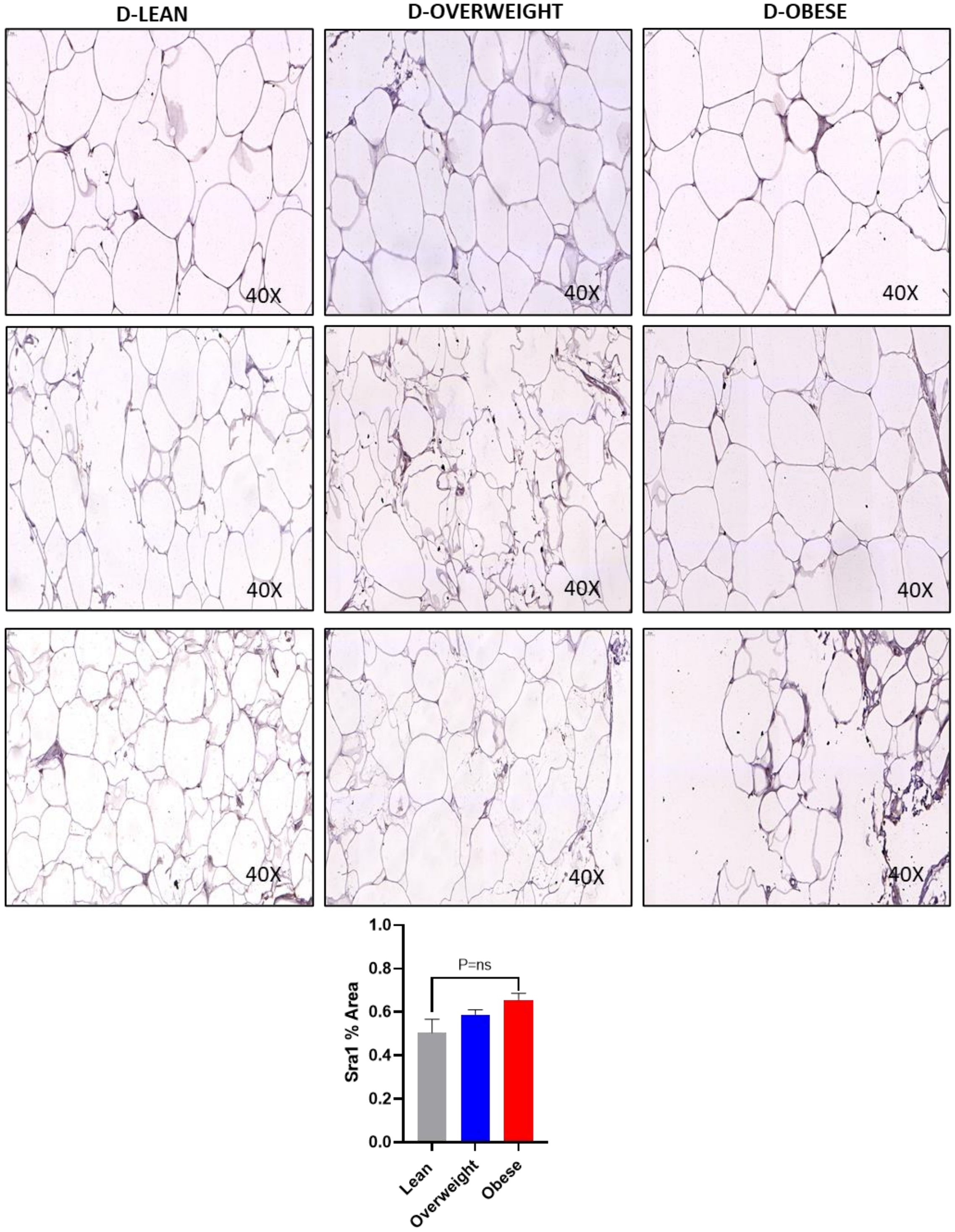

Figure S1. Increased SRA1 protein expression in obese adipose tissue. Adipose SRA1 protein expression was determined by immunohistochemistry (IHC) in 5 lean, 5 overweight, and 5 obese individuals. The representative images obtained from five independent determinations with similar results show elevated adipose SRA1 protein expression in overweight and obese individuals compared with lean: (A) 40× magnification of IHC images.

TNF-α protein expression in the adipose tissue of individuals without diabetes

A

ND-LEAN | **ND-OVERWEIGHT** | **ND-OBESE**

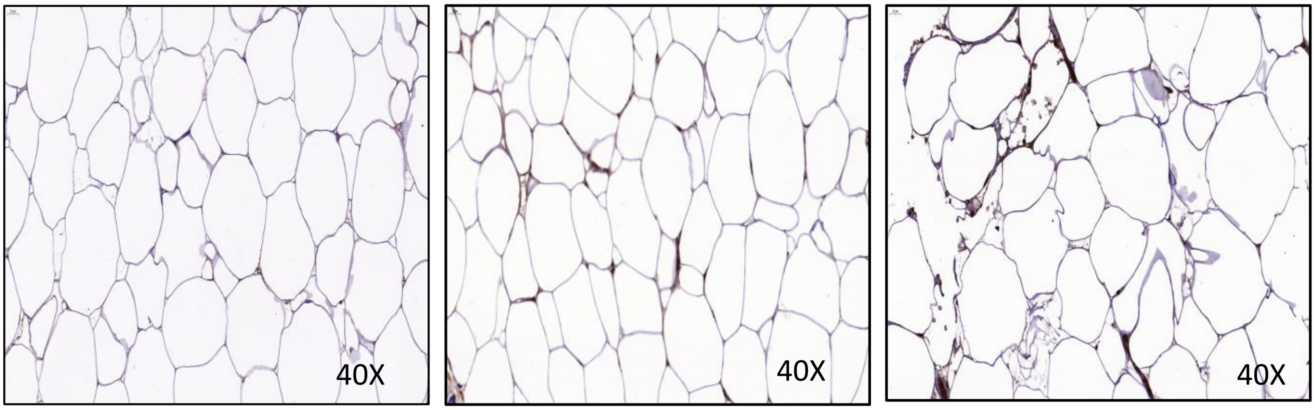

B **IL-18 protein expression in the adipose tissue of individuals without diabetes**

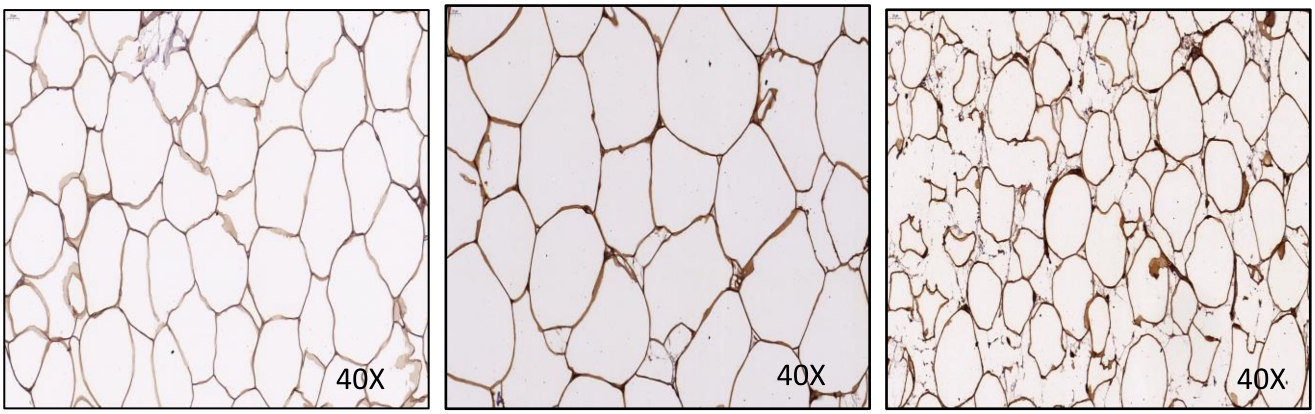

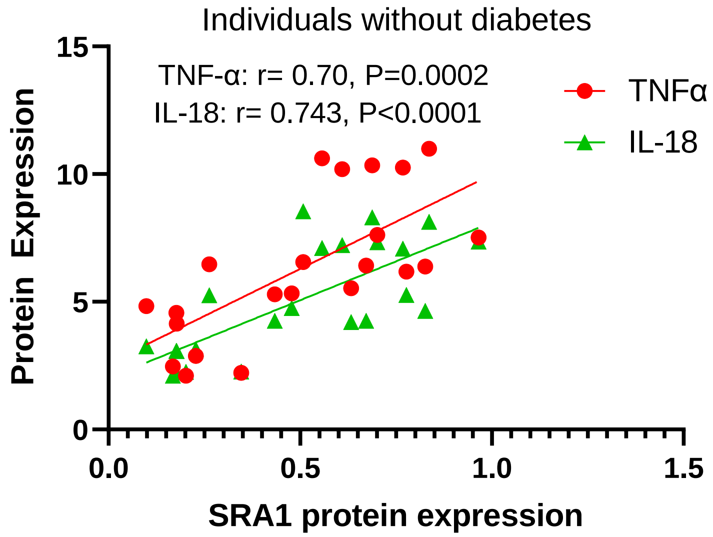

Figure S2. Increased TNF-α and IL-18 correlated with SRA1 protein expression in adipose tissue. Adipose TNF-α , IL-18 and SRA1 protein expression was determined by immunohistochemistry (IHC) in 8 lean, 6 overweight, and 9 obese individuals. The representative images obtained from five independent determinations: (A) 40× magnification of IHC images. Correlation between SRA1 protein expression with inflammatory markers TNF-α and IL-18.

A TNF-α protein expression in the adipose tissue of individuals with diabetes

D-LEAN D-OVERWEIGHT D-OBESE

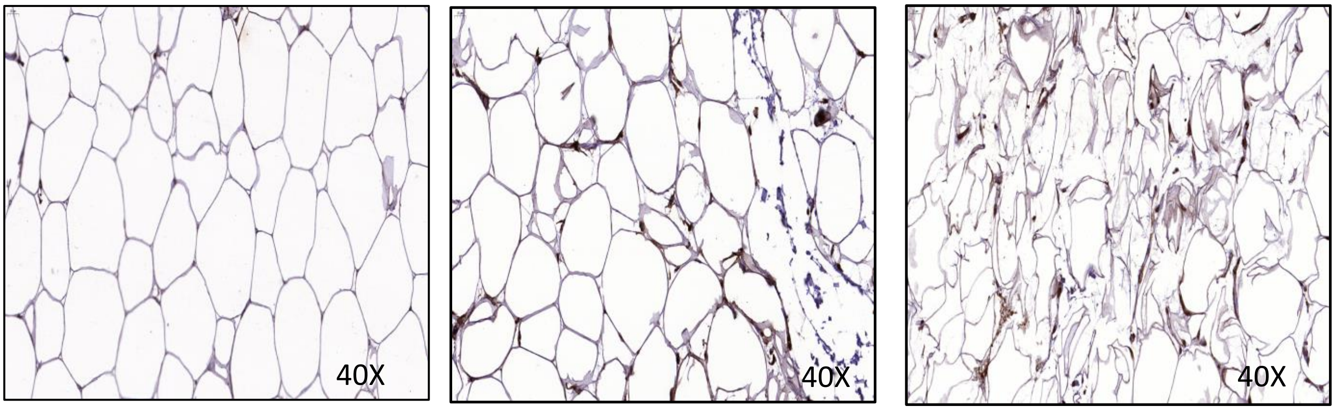

B IL-8 protein expression in the adipose tissue of individuals with diabetes

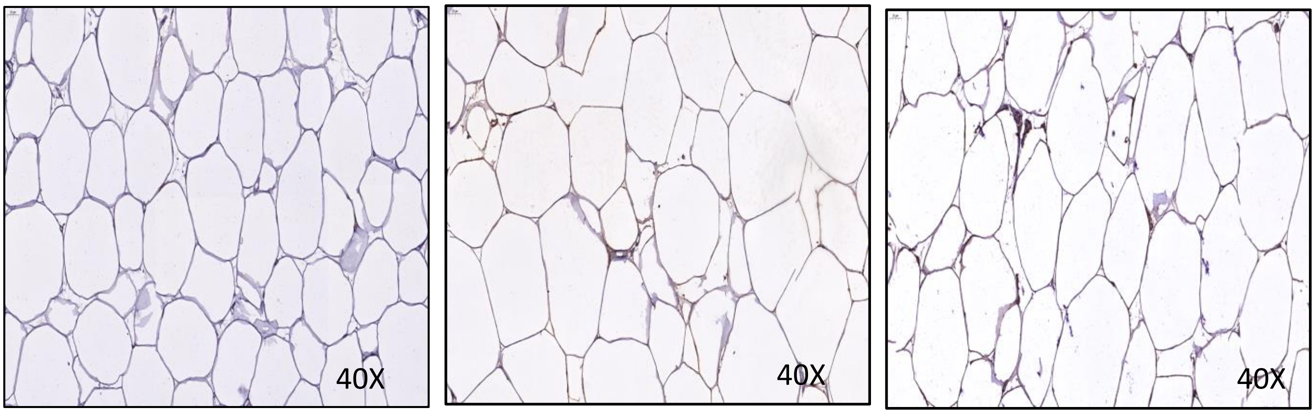

C CCL2 protein expression in the adipose tissue of individuals with diabetes

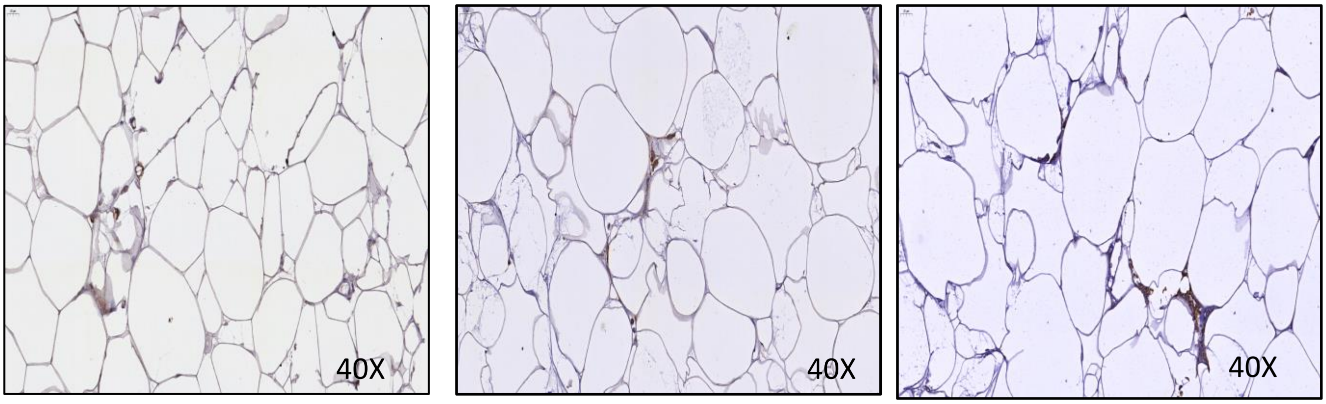

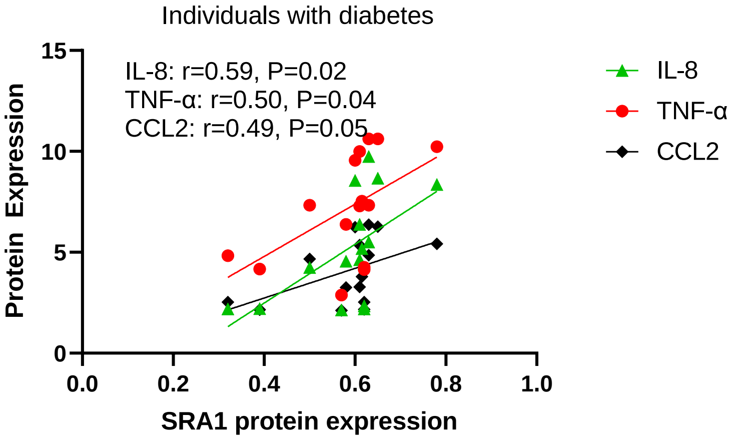

Figure S3. IL-8, TNF-α and CCL2 protein expression in correlated with SRA1 protein expression in adipose tissue. Adipose IL-8, TNF-α , CCL2 and SRA1 protein expression was determined by immunohistochemistry (IHC) in 5 lean, 5 overweight, and 5 obese individuals with diabetes. The representative images obtained from five independent determinations: (A) 40× magnification of IHC images. Correlation between SRA1 protein expression with inflammatory markers IL-8, TNF-α and CCL2.

Material and methods for immunohistochemistry

Paraffin-embedded sections (4µm thick) of subcutaneous adipose tissue were deparaffinized in xylene and rehydrated through descending grades of ethanol (100, 95, and 75 %) to water. Antigen retrieval was performed by placing slides in target retrieval solution (pH 6.0; Dako, Glostrup, Denmark) in the pressure cooker boiling for 8 min and cooling for 15 min. After washing in PBS, endogenous peroxidase activity was blocked with 3 % H2O2 for 30 min and non-specific antibody binding was blocked with 5 % nonfat milk for 1 h followed by 1 % bovine serum albumin solution for 1 h. The slides were incubated at room temperature overnight with primary antibody (1:400 dilution of rabbit polyclonal anti-Sra1 antibody; ThermoScientific PA5-62145; anti-TNfα Abcam ab9635; anti-IL18 Abcam ab191152; IL8 Abcam ab106350; anti-CCL2 ThermoScientific PA5-80413S). After washing with PBS (0.5 % Tween), slides were incubated for 1 h with secondary antibody (goat anti-rabbit conjugated with horseradish peroxidase (HRP) polymer chain; EnVision™ Kit from Dako Code K4063, Glostrup, Denmark) and color was developed using 3,3'-diaminobenzidine (DAB) chromogen substrate. Specimens were washed in running tap water, lightly counterstained with Harris hematoxylin, dehydrated through ascending grades of ethanol (75, 95, and 100 %), cleared in xylene, and finally mounted in dibutylphthalate xylene (DPX). For analysis, digital photomicrographs of eight different regions to assess the regional heterogeneity in tissue samples were taken in 20X using PannoramicScan (3DHistech, Hungry). All samples were analysed using imageJ software (NIH,USA).

Table 1. Correlation of SRA1 expression level with various clinical and biochemical markers.

	Non-Diabetic						Diabetic					
	Lean (n=8)		Overweight (n=19)		Obese (n=28)		Lean (n=4)		Overweight (n=13)		Obese (n=36)	
	r	*p*	*r*	*p*	*r*	*p*	*r*	*p*	*r*	*p*	*r*	*p*
**Age**	0.359	0.379	0.293	0.224	-0.074	0.707	0.400	0.750	-0.114	0.711	0.116	0.500
**Weight**	0.476	0.243	0.141	0.564	-0.210	0.284	1.000	0.083	0.220	0.470	-0.066	0.701
**Height**	0.405	0.327	0.086	0.726	-0.335	0.081	0.633	0.500	0.325	0.277	-0.221	0.195
**BMI**	0.191	0.665	0.221	0.363	0.123	0.532	0.800	0.333	0.165	0.587	0.119	0.491
**PBF**	0.527	0.237	0.209	0.419	0.005	0.980	1.000	0.333	-0.064	0.854	-0.115	0.559
**Waist**	0.071	0.906	-0.083	0.749	-0.140	0.515	0.500	0.999	0.694*	0.022	-0.026	0.897
**Hip**	0.607	0.167	0.169	0.515	0.064	0.771	0.500	0.999	-0.546	0.088	0.055	0.781
**WHR**	-0.357	0.444	-0.062	0.822	0.332	0.114	-0.500	0.999	-0.055	0.881	0.131	0.524
**GLU**	0.228	0.589	-0.066	0.788	0.069	0.726	-0.400	0.750	-0.270	0.394	0.030	0.862
**TGL**	-0.095	0.840	0.364	0.126	0.004	0.985	-0.400	0.750	-0.182	0.573	0.002	0.989
**Chol**	0.262	0.536	-0.070	0.775	0.070	0.725	-0.800	0.333	-0.032	0.926	-0.066	0.704
**HDL**	-0.286	0.501	-0.025	0.921	0.256	0.189	-0.400	0.750	0.133	0.683	0.168	0.335
**LDL**	0.145	0.735	-0.277	0.252	0.006	0.976	-0.633	0.500	-0.098	0.761	-0.017	0.926
**HbA1c**	0.575	0.143	-0.254	0.310	0.218	0.266	-0.400	0.750	-0.741	0.008	-0.220	0.197
**Insulin**	-0.119	0.793	0.429	0.087	0.282	0.307	-1.000	0.333	0.139	0.707	0.095	0.607
**HOMA-IR**	0.048	0.935	0.316	0.216	0.386	0.157	-1.000	0.333	0.164	0.657	0.134	0.465

BMI, body mass index; PBF, Percent Body Fat, WHR, Waist/Hip ratio; GLU, fasting plasma glucose, TGL, plasma triglycerides; Chol, cholesterol; HDL, high-density lipoprotein; LDL, low-density lipoprotein; HbA1c, glycated hemoglobin; HOMA-IR, homeostatic model assessment. *p<0.05, and **p<0.01.
